# Prognostic Value of Ki67 and Other Clinical and Histopathological Factors in Canine Apocrine Gland Anal Sac Adenocarcinoma

**DOI:** 10.3390/ani11061649

**Published:** 2021-06-02

**Authors:** Emanuela Maria Morello, Marzia Cino, Davide Giacobino, Arturo Nicoletti, Selina Iussich, Paolo Buracco, Marina Martano

**Affiliations:** 1 Department of Veterinary Sciences, University of Torino, Largo Braccini 2, 10095 Grugliasco, Italy; emanuela.morello@unito.it (E.M.M.); davide.giacobino@unito.it (D.G.); arturo.nicoletti@unito.it (A.N.); selina.iussich@unito.it (S.I.); paolo.buracco@unito.it (P.B.); 2 Department of Medical-Veterinary Science, Univesrity of Parma, Strada del Taglio 10, 43126 Parma, Italy; marina.martano@unipr.it

**Keywords:** dog, anal sac adenocarcinoma, AGASACA, Ki67, prognosis

## Abstract

**Simple Summary:**

Apocrine gland anal sac adenocarcinoma (AGASACA) is a locally aggressive tumor with a high metastatic rate to the regional ileo-sacral lymph nodes and later to distant sites. Clinical signs depend on the tumor and regional lymph nodes size or on the paraneoplastic hypercalcemia. Therefore, dogs are often referred for perineal swelling, tenesmus, constipation and/or polyuria, polydipsia. Surgical excision of both the neoplastic anal sac and the metastatic regional lymph nodes represents the standard of care. Adjuvant chemotherapy and/or radiotherapy are also indicated. Prognostic factors include tumor size, hypercalcemia, regional lymph nodes metastasis and tumor histologic features. The aim of the study was to retrospectively evaluate the prognostic significance of tumor Ki67 expression. The authors’ hypothesis was that higher Ki67 index correlated with decreased disease-free interval and overall survival time. Clinical data such as tumor size, regional lymph nodes metastasis and hypercalcemia at presentation and histological features such as tumor pattern, mitotic count, necrosis, inflammatory infiltration, vascular invasion, anisokaryosis, and anisocytosis were also investigated and correlated to the oncologic outcome in the dogs included in the study.

**Abstract:**

Apocrine gland anal sac adenocarcinoma (AGASACA) is locally aggressive and highly metastatic to regional lymph nodes. The aim of this study was to evaluate the prognostic significance of Ki67 in surgically excised AGASACA. Prognostic impact of size, regional lymph nodes metastasis, hypercalcemia, histologic pattern, mitotic count, necrosis, inflammatory and lympho-vascular invasion, anisokaryosis and anisocytosis was also evaluated. Thirty-five dogs were included, twenty-four of which also had metastatic lymph nodes. When the entire population was evaluated, only metastatic disease spread to regional lymph nodes, and necrosis and inflammatory infiltration were correlated to prognosis. When only dogs with metastatic disease were evaluated, size, solid histologic pattern, presence of lymphatic and vascular invasion showed influence on prognosis. Ki67 index was not associated with survival time and disease free interval in any case. The results of this study showed that lymph nodes metastasis at diagnosis reduced disease free interval. Moreover, tumor size greater than 5.25 cm, presence of lymphatic and vascular invasion and a solid histologic pattern were associated with a shorter survival time in dogs with metastasis to regional lymph nodes. Ki67 expression was not significantly associated with prognosis, therefore it could not be considered as a prognostic factor in this tumor type, while the role of hypercalcemia remained unclear.

## 1. Introduction

Apocrine gland anal sac adenocarcinoma (AGASACA) is a relative uncommon epithelial neoplasm of the anal sacs, representing 2% of all skin tumors and 17% of all perianal malignancies in dogs [1,2]. Generally, it occurs in older animals without any breed prevalence; although a genetic predisposition has been described for the English Cocker Spaniel [3,4]. Although several early studies described AGASACA as a disease condition of older females, recent and larger reports showed a similar sex occurrence [2,3,5,6,7,8,9,10]. 

AGASACA is a locally aggressive tumor with a high potential to metastasize via lymphatics to the regional ileo-sacral lymph nodes and later to distant sites, such as spleen, liver, lungs and bone. Metastatic disease is reported in between 26% and 89% of cases at the time of diagnosis [3,7,8,9,10,11,12]. Dogs can display a variety of clinical signs related to tumor size (perineal swelling, pain, scooting), regional lymph nodes enlargement (tenesmus, constipation) or to paraneoplastic hypercalcemia (polyuria, polydipsia, lethargy). As reported in previous studies, the incidence of this paraneoplastic syndrome is between 16% and 53% [7,8,9,13], and there is some conflicting information regarding its prognostic role. According to some authors [6,9,13], its development negatively impacts survival time, whereas others reported no statistical difference in survival time between hypercalcemic and normocalcemic dogs [7,10,11,14,15].

Several studies described a significant association between tumor size and outcome, but different cut offs were proposed, making the identification of a unique significant prognostic value difficult to determine [9,11,12,14,15,16,17,18]. In one study, dogs with AGASACA <10 cm^2^ survived 584 days while those with AGASACA >10 cm^2^ survived only 292 days [9]. Recently, it was reported that dogs with tumors larger than 2.5 cm were significantly more likely to present metastatic disease [18].

The presence of regional lymphadenopathy at the time of presentation is correlated with a worse prognosis in the majority of the studies [10,11,12,14].

The treatment for AGASACA involves both the local control of the primary tumor and of the regional metastasis. When feasible, surgical excision of the primary tumor and lymph nodes represents the mainstay of the treatment, while the role of chemotherapy and radiotherapy is still unclear [7,8,9,10,11,12,16,19,20,21,22,23,24]. Median survival time (ST) after surgical excision ranges between 386 and 960 days [7,8,9,10,11,12,14,15,19,25], but a recent study reported a median ST of 1237 days in dogs with non-metastatic AGASACA treated with surgery alone [17]. The disease-free interval (DFI) ranged between 262 and 443 days [10,11,17]. 

Prognostic factors include tumor size [9,12,14,18], hypercalcemia at presentation [6,9,13], regional lymph nodes metastasis [10,11,12,14] and tumor histologic pattern [14,26]. The prognostic significance of other histological features, such as peripheral infiltration, cellular pleomorphism, mitotic count, presence of necrosis and presence of peritumoral lympho-vascular invasion, have also been investigated in recent studies [14,17,18].

Ki67 is a nonhistone nuclear protein expressed at every stage of the cell cycle (G1-M) but not in resting cells (G0); its expression is considered a reliable tumor proliferative marker [27,28]. The Ki67 index has been used prognostically in different canine tumors, such as malignant oral melanoma, mast cell tumors, mammary tumors and diffuse large B-cell lymphoma [29,30,31,32,33].

To our knowledge, only one study evaluated the association between Ki67 expression and oncologic outcome in 20 dogs with non-metastatic AGASACA with a tumor size <3.2 cm but no significant results have been reached [17].

AGASACA has been classified into three different histological patterns (solid, rosette and tubular type) [26,34], but in a recent study [14] a new pattern of classification has been proposed (solid, tubules/rosettes/pseudorosettes and papillar) showing a worse outcome for AGASACA with a predominant solid growth, as previously reported by Suzuki et al. (2013) [26].

The primary aim of this study was to retrospectively evaluate the prognostic significance of Ki67 expression for surgically excised AGASACA and to find a reliable Ki67 threshold value for non-computerized diagnostic immunohistochemistry, using two counting methods already reported in the literature. The authors’ hypothesis was that higher Ki67 index correlated with decreased DFI and overall ST.

The second aim was to determine if the size of the primary tumor, the presence of regional lymph nodes metastasis and hypercalcemia at presentation, were predictive of outcome in dogs surgically treated, with or without of adjunctive chemotherapy.

The prognostic impact of histological factors such as histologic pattern, mitotic count, necrosis, inflammatory infiltration, lympho-vascular invasion, anisokaryosis, anisocytosis on outcome were also evaluated.

## 2. Materials and Methods

In this retrospective study, client-owned dogs surgically treated for AGASACA at the Veterinary Teaching Hospital (VTH) of Grugliasco of the University of Torino, between September 2011 and November 2019, were included.

### 2.1. Selection Criteria

Dogs were included if they underwent a surgical excision of the primary tumor and of regional lymph nodes when enlarged, with or without adjuvant chemotherapy. For each animal, the histologic evaluation of the entire primary tumor and of the excised regional lymph nodes, as well as the availability of the hematoxylin and eosin (H&E) histology slides and of the paraffin tissue blocks for immunohistochemistry represented an additional inclusion criterion.

Dogs with distant metastasis identified during a pre-surgical computed tomographic (CT) staging and those affected by concurrent illnesses that could significantly reduce the survival time were excluded from the study. A minimum follow-up of 1-year post-surgery was required; and information was updated up to 31 December 2020.

### 2.2. Data Collection

Information retrieved from the database of the Grugliasco VTH included signalment (breed, sex, age and weight), primary tumor side, clinical signs at presentation, presence of hypercalcemia [defined as an ionized calcium >1.45 mmol/L based on the reference range (1.25–1.45 mmol/L) of the internal laboratory], presence or absence of ileo-sacral lymphadenopathy by digital rectal examination and CT scan evaluation, and complete blood analyses.

Before surgery, all dogs underwent pre- and post-contrast whole body CT to detect enlarged regional lymph nodes and distant metastasis and to identify the extension of the primary perianal mass.

Date of surgery, surgical complications, size of the excised mass defined as the longest dimension evaluated on formalin fixed tissue, adjuvant chemotherapy, date of tumor progression defined as local recurrence and/or regional lymph nodes and distant metastasis, disease free interval (DFI) defined as the time between surgery and local recurrence or metastatic spread, survival time (ST) considered as the time from surgery until the death of the dog from any cause and causes of death were recorded. Post-surgical complications and wound status were monitored until skin sutures removal (usually 10 to 15 days after surgery). Long term follow up was obtained for all patients by telephone contact with owners or referring veterinarians.

A written consent was obtained from the owners for the anesthetic, diagnostic, histological and surgical procedures before including the animal. Owners signed an informed consent for the procedure as a standard procedure for the treatment of the disease. No specific informed consent statement was signed for the inclusion in this retrospective study. Peri-operative standard-of-care management, including analgesia, was assured for all dogs. The study does not fall within the application areas of Italian Legislative Decree 26/2014 which governs the protection of animals used for scientific or educational purposes. Therefore, ethical approval is waived for this study. Animals were not treated as part of an experimental study but out of necessity, and only the data needed for the study purposes were later selected.

### 2.3. Histological Analysis

Hematoxylin and eosin glass slides were jointly reviewed by 2 veterinary pathologists (S.I. and A.N.) who were blinded to the clinical data, by means of a multiheaded microscope (NIKON Y-THM, Japan 0120983). Diagnosis of AGASACA was confirmed and histopathological features of each tumor were described; in particular, evaluation of the predominant tumor pattern, necrosis, inflammatory infiltration, peripheral neoplastic infiltration, lymphatic invasion, cellular pleomorphism (anisokaryosis and anisocytosis) and mitotic count were considered.

Histological pattern of lymph node metastasis was also evaluated.

The different patterns of neoplastic cells arrangement were grouped as: solid, rosettes/tubules and papillary. The pattern was considered *solid* when the neoplastic cells were closely packed, supported with minimal stroma and arranged in sheets, lobules or rarely in nests. The *rosette/tubules* pattern was assigned when neoplastic cells were radially arranged around a central hub composed either of an empty-appearing lumen (tubule) or a space filled with cytoplasmic processes (rosettes). Tubules and rosettes were often present concurrently and, when the central lumens were compressed, the distinction between the two was difficult; for this reason, they were grouped in the same category. In the *papillary* pattern, the neoplastic cells were arranged in one or multiple layers disposed around a fibrovascular stalk, forming arborescent projections. For each tumor, the predominant histologic pattern was attributed when representative of more than 50% of the tumor [14].

Necrosis, intratumoral inflammatory infiltration, lympho-vascular invasion (defined as the presence of tumor cells within a definite endothelial-lined space, lymphatics or blood vessels) were classified as binary variables (present or absent) [14].

The extent of cellular pleomorphism was assessed examining 10 high-power fields (HPF) of 0.237 mm^2^ at 400× magnification, and areas of high pleomorphism were selected. Specifically, variability in cell morphology (anisocytosis) or nuclear morphology (anisokaryosis) were considered. Both were categorized as minimal, if there was a fairly uniform shape and size, moderate, when a more marked variation in nuclear or cell morphology was recorded. Nuclear and cellular pleomorphism was described as marked when a more pronounced variation in nuclear shape and size was observed.

Mitotic count (MC) was calculated as the absolute number of mitoses counted in 10 consecutive, nonoverlapping HPF of 0.237 mm^2^ at 400× magnification; areas of highest mitotic activity were selected [35]. Areas of extensive necrosis and apoptotic or pyknotic nuclei were excluded.

### 2.4. Immunohistochemistry

Four μm sections of formalin fixed paraffin embedded (FFPE) tissue were cut, mounted on positively charged slices (Menzel- Gläser Superfrost plus. Thermo scientific), deparaffinized in xylene, rehydrated in graded ethanol and rinsed in distilled water. Endogenous peroxidases were blocked by incubating sections in 0.3% hydrogen peroxide diluted in distilled water for 30 min.

Antigens retrieval was performed placing sections in a microwave-resistant container with a solution of tris-EDTA (pH 9.0) and heating 3 times (3 min each) at 700 W. The sections were left to cool at room temperature for 15 min and were next immerged in phosphate buffer saline solution (pH 7.4) for 5 min.

Tissue sections were subsequently incubated with Blocking serum (Vector^®^ Vectastain Elite ABC kit) for 20 min in a humidified chamber, then incubated with the monoclonal mouse anti-human Ki-67 antibody (clone MIB-1, M7240, Dako, Glostrup, Denmark) diluted 1:200 in a PBS solution, in a humidified chamber for 120 min.

After incubation, tissue sections were washed in PBS-tween20 diluted 1:1000 and then incubated with biotinylated secondary antibody (Vector^®^ Vecstain Elite ABC kit, Anti-Mouse IgG/Rabbit IgG) in a humidified chamber, for 30 min.

Avidin-biotin system (Vecstain^®^) was used for immunolabeling, and reactions were visualized with 3′3′-diaminobenzidine (DAB, Peroxidase Substrate Cat. No. SK-4100). At the end, slides were counterstained with Mayer’s hematoxylin, dehydrated, and mounted for evaluation by light microscopy. 

### 2.5. Evaluation of Ki67

Ki67 positive cells were evaluated using standard light microscopy and two manual counting techniques, previously described [17,29]. In both cases, areas of necrosis and inflammation were avoided and lymphocytes were used as positive controls. The whole slides were evaluated at low power magnification (10×) in order to identify areas in which the staining was most evident. Firstly, the Ki67 index was calculated as the average of 3 nonconsecutive HPF of 0.237 mm^2^ at 400× magnification in which 100 consecutive cells were counted. Secondly the Ki67 index was calculated as the mean number of labeled neoplastic cells in 5 HPF [29]. 

### 2.6. Statistical Analysis

The analyses were performed using GraphPad Prism (version 9.0.0 for Windows, GraphPad Software, San Diego, CA, USA, www.graphpad.com) and a *p*-value < 0.05 was considered statistically significant.

Descriptive statistics were provided for age, bodyweight, size of primary tumor, Ki67 and mitotic count (mean, median and range). Normal distributions were checked graphically using the Shapiro Wilk test.

The overall median DFI and ST were estimated using the Kaplan–Meier method. Spearman correlation was used to find a correlation between lymph nodes Ki67 index and anal sacs Ki67 index (for both counting methods). Because of the resulting high correlation, the mean value of Ki67 for each patient [(lymph nodes Ki67 + anal sacs Ki67)/2] was calculated and used in the descriptive analyses. The following factors were evaluated for association with ST and DFI using Kaplan–Maier curves and long-rank test: presence of lymph nodes metastasis, presence of hypercalcemia, tumor maximum dimension (longest dimension evaluated on formalin fixed tissue), type of treatment, median Ki67 index, MC, histological pattern, necrosis, inflammatory infiltration, lympho-vascular invasion, anisokaryosis and anisocytosis.

Fisher exact test was used to compare the presence of lymph nodes metastasis at the time of presentation and DFI and ST.

## 3. Results

### 3.1. Signalment

Thirty-five client-owned dogs were included in the study. Twenty-six (74.29%) dogs were female (4 intact and 22 spayed) and nine (25.71%) were male (6 neutered and 3 intact). Mean and median age was 10.93 and 11 years, respectively (range 7–15). Mean and median bodyweight was 23.96 and 24 Kg, respectively (range 7–40). There were 17 mixed-breed dogs, 7 Labrador Retrievers, 3 Border Collies, 2 German Shepherds and one each for the following breeds: Basset-Hound, Beagle, Boxer, Cavalier King Charles Spaniel, Golden Retriever and Siberian Husky (Appendix A, Table A1).

### 3.2. Clinical Presentation and Staging

In eight cases (22.86%) the AGASACA was an incidental finding, while the other 27 dogs (77.14%) had one or more of the following clinical symptoms: tenesmus, excessive perineal grooming, limb weakness, pain to abdominal palpation, abnormal stool shape. Polyuria and polydipsia were present in eight dogs (22.86%), seven of which (87.5%) were hypercalcemic. Digital rectal examination was performed in all dogs. Seventeen (48.57%) dogs had a clinically palpable ileo-sacral lymphadenopathy, confirmed by CT scan; histological evaluation of the surgically excised lymph nodes confirmed the metastatic spread.

A cytological diagnosis of AGASACA was obtained in 18 out of 35 dogs by a trans-cutaneous fine-needle aspiration of the mass performed during the clinical evaluation at the VTH; in 17 cases the cytology was performed by the referring veterinarian. In all cases, cytological diagnosis was subsequently confirmed by histopathology after surgical excision of the tumor.

A CBC, serum biochemical and blood gas analysis were performed in all dogs at the time of initial diagnosis. Hypercalcemia was found in 12/35 (34.29%) dogs; all became normocalcemic after surgery. No other abnormalities were detected on the blood work.

Preoperative total-body CT scan was performed in all dogs. Lymphadenomegaly was diagnosed in 24 dogs (68.58%). No evidence of distant metastatic disease was detected on CT scan in any of the 35 dogs (Appendix A, Table A1).

### 3.3. Treatment

Seventeen (48.57%) and 12 dogs (34.29%) underwent a right and left anal sacculectomy, respectively. Six (17.14%) dogs underwent bilateral sacculectomy, but histology only confirmed bilateral AGASACA in 2 cases, while 3 dogs had a left-side tumor and one dog a right-side tumor (Appendix A, Table A1).

In 3 of the 35 dogs undergoing surgery, half of the external anal sphincter was excised because of the primary tumor involvement. However, in none of these dogs fecal incontinence occurred; 1 of them had anal stenosis that required a further surgery to allow a easier fecal transit. 

Eleven (31.42%) dogs underwent only anal sacculectomy. Twenty-four dogs with lymphadenomegaly underwent combined ventral midline celiotomy for lymph node extirpation and primary tumor excision during the same anesthetic episode. In no case a pelvic osteotomy was necessary. In two (8.3%) of 24 dogs, lymph nodes were firmly attached to iliac vessels, thus surgeons decided to perform only a debulking procedure and in these cases, DFI was considered equal to 0 days.

Adjuvant chemotherapy was proposed in all cases and accepted by the owners of 23 dogs (65.72%); 18 of which had regional lymph nodes metastasis at presentation, and 5 dogs with non metastatic anal sac tumor.

Toceranib phosphate (at standard dosage of 2.75 to 3.25 mg/Kg PO on Monday, Wednesday and Friday) (Palladia^®^, Zoetis Italia S.r.l., Roma, Italy) was used in 21/23 dogs (91.67%). The median duration of toceranib treatment was 180 days (range 30–598 days). One dog received carboplatin (at a dose of 300 mg/mq every 3 weeks as an IV infusion over 10 min for four cycles) (Carboplatino^®^, Teva, Italia S.r.l., Roma, Italy), 1 dog was initially treated with melphalan (3 mg/mq daily PO for 4 months) (Alkeran^®^, Glaxo Smith Kline, Aspen Italia S.r.l., Verona, Italy). In case of tumor progression, dogs received chemotherapy, or a new surgical excision plus chemotherapy, or metronomic chemotherapy (cyclophosphamide 12.5 mg/mq q24h PO (Endoxan Baxter^®^, S.p.A., Roma, Italy), piroxicam 0.3 mg/Kg q24h (Feldene^®^, Pfizer, Italia S.r.l., Roma, Italy), thalidomide from 3 to 6 mg/kg q24h PO) or were euthanized. Data regarding adjuvant treatment are summarized in Table 1.

### 3.4. Follow Up

Median ST for all treated dogs was 877 days (range 108–1434); median DFI was 474 days (range 0–1434).

Median ST and median DFI for dogs that underwent surgical excision only were 559 and 523 days, respectively. Median ST and DFI for dogs that also received adjuvant chemotherapy were 946 and 378 days, respectively (Appendix A, Table A1).

Eighteen of 24 dogs that had lymph nodes involvement at the time of diagnosis, were treated with chemotherapy. These dogs had a median ST of 877 days and a median DFI 283 days. Six dogs underwent only surgical excision of AGASACA. These dogs had a median ST and a median DFI of 465 and 253 days, respectively.

At the end of the study period, three (8.58%) dogs were still alive, two of which were free of disease after 819 and 385 days, respectively, and one was diagnosed as experiencing disease progression after 913 days.

Thirty-one (88.57%) dogs died or were euthanized, 18 of which (58.06%) because of AGASACA-related causes. One (2,85%) dog was lost to follow up 598 days after surgery, with no signs of local o distant tumor relapse at the last telephone contact with owner (Appendix A, Table A1).

Thirteen dogs (37.14%) died for unrelated tumor causes. The cause of death was hemangiosarcoma (one dog), heart failure (two dogs), sudden death (three dogs), metastatic mammary tumor (one dog) and multicentric lymphoma (one dog), while two dogs were euthanized because of suspected neurologic problems and three dogs because of diseases related to old age, as reported by owners. A necropsy was not permed in any case.

Thirteen (37.14%) dogs did not show any evidence of recurrence, while twenty-two dogs (62.86%) experienced disease progression, 11 of which received additional treatments (four and seven dogs were treated with surgery and with chemotherapy, respectively), while 11 dogs were euthanized. Of these 22 dogs, 17 had lymph nodes metastasis at presentation, and five had only the anal sac tumor (one dog with bilateral AGASACA, one with a left AGASACA and three dogs with a right side AGASACA) (Table 2).

Tumor progression included: both local recurrence and regional lymph nodes metastasis in two dogs, regional lymph-nodes metastasis only in 15 dogs and distant metastasis in five dogs. Distant metastatic sites included spleen (*n* = 2), periportal lymph nodes (*n* = 1), liver (*n* = 1) and lumbar vertebrae (*n* = 1).

### 3.5. Histological Data

Histological features of the 35 AGASACA tumors are summarized in Table 3.

### 3.6. Prognostic Factors

Clinical factors (tumor size, hypercalcemia and presence of regional lymph nodes metastases at the time of diagnosis) and histological features (Ki67 index, tumor pattern, necrosis, inflammatory infiltration, lymph vascular invasion, cellular pleomorphism) were investigated on the entire population (*n* = 35 dogs) and were correlated to ST and DFI in order to find a prognostic significance.

Mean and median size of the primary tumor measured after formalin fixation (maximum dimension) was 4.11 and 4 cm, respectively (range 0.5–10 cm). The median size of the primary tumor was correlated to the ST and DFI, but no significant differences were found between dogs with tumor <4 cm and ≥4 cm (median ST 877/946 days, *p* = 0.24; median DFI 385/913 days, *p* = 0.95). As previously described by Pradel et al. [14], tumor size was divided into quartiles and the third was selected as a cut-off (6 cm). Dogs with tumor <6 cm had longer median ST than dogs with a mass ≥6 cm (median ST 946 vs 352 days *p* = 0.02), but no significant association was found when comparing the two groups for the median DFI (*p* = 0.21).

There was not significant difference in ST (*p* = 0.054) and DFI (*p* = 0.40) between hypercalcemic (ST = 462.5 days and DFI= 329 days) and normocalcemic dogs (ST = 1094 days and DFI = 576 days).

Dogs with lymph nodes metastasis had a significantly shorter median DFI (283 days) than dogs without lymph nodes metastasis (940 days) (*p* = 0.02), and there was a survival advantage for dogs without lymph nodes metastasis (median ST 1237 vs 545 days), but this difference was not significant (*p* = 0.1).

Although the histological features described in Table 3 were correlated to ST and DFI of the entire population (35 dogs), only the presence of lympho-vascular invasion was statistically significant: median ST 385/1237 days (*p* = 0.003); median DFI 376/918 days (*p* = 0.03). Moreover, cases with absence of necrosis had a median ST (946 days) significantly longer (*p* = 0.02) than cases with presence of necrosis (352 days).

The overall median Ki67 index for the 35 dogs, calculated for 300 immunoreactive cells in 3 nonconsecutive HPF was 34.33% (range 19.6–55.98; mean 34.58%). Using the counting method described for malignant oral melanoma [29], the median percentage of Ki67 for 500 cells was 31.9% (range 16.20–51.20; mean 31.42). Neither the Ki67 index threshold of 34.33% was significantly associated with median ST (Ki67 < 34.33 = 946 days; Ki67 ≥ 34.33 = 877 days; *p* = 0.89) and median DFI (Ki67 < 34.33 = 523; Ki67 ≥ 34.33 = 376 days; *p* = 0.92), nor did the Ki67 cut-off of 31.9% (median ST 946 vs 545 days; *p* = 0.29 and median DFI 576 vs 376 days; *p* = 0.33) (Appendix A, Table A1).

Because of the marked difference in ST and DFI between the two groups (dogs with AGASACA only and those with AGASACA and metastases to regional lymph nodes), the authors decided to investigate a series of potential prognostic factors only in dogs with metastatic lymph nodes (Table 4).

Referring to clinical data on the AGASACA population with metastases to the regional lymph nodes (*n* = 24), mean, median size and third quartile of the primary tumor after surgical excision were 3.96, 3.0 and 5.25 cm (range 0.5–10 cm), respectively. The median tumor size value of 3 cm and the third quartile value of 5.25 cm were used for further correlations (Table 4).

Dogs with metastatic regional lymph nodes and primary tumor size <5.25 cm showed a longer median ST than those with primary AGASACA >5.25 cm (median ST 877 vs 220.5; *p* = 0.005), but no significant association was found between this cut-off and DFI (*p* = 0.11).

The development of hypercalcemia lead to a marked reduction in survival, but, as previously seen in the entire population, this value (*p* = 0.054) is not statistically significant, although very close.

Referring to the histologic data on the AGASACA population with metastases to the regional lymph nodes (*n* = 24), given the low number of cases for each histotype different from the solid, the predominant tumor pattern was divided into two groups (solid vs. other types); this classification was significantly associated with DFI (*p* = 0.03) but not with ST (*p* = 0.32).

Median MC <16.5 was very close to significance (*p* = 0.053) and 12 dogs with median mitotic count <16.5 apparently lived more than others.

The presence of lymphatic and vascular invasion significantly reduced survival time (median ST 366 days vs 1120 days; *p* = 0.02).

Ki67 index, anisocytosis and anisokaryosis, MC, inflammatory invasion, and necrosis were not significantly associated with ST and DFI.

## 4. Discussion

In agreement with early studies that described AGASACA as a condition of older bitches [2,5,6], 74.29% of dogs in this study were female (4 intact and 22 spayed) with a mean age of 10.31 years. Body weight and breeds were consistent with the literature [7,8,9,18], but English Cocker Spaniels dogs were not present in this study, although this breed seemed to be the more commonly affected in several publications from the United Kingdom [3,12,15,25], and in which a genetic predisposition has been described [4].

Clinical signs were similar to that described in the literature [9,10,11]. In 22.86% of cases (eight dogs) AGASACA was an incidental finding during physical examination. This incidence is lower if compared to that reported by Williams et al. (2003), in which the tumor was incidentally found in 39% of 113 dogs [9].

In the present study, AGASACA was bilateral in 2 of 35 patients (5.7%); no dogs showed the occurrence of contralateral anal sac tumor after being treated for a monolateral form. This incidence is lower than that reported in other studies (2.9–6%) [25,36].

The primary aim of the present study was to investigate the Ki67 prognostic role in surgically excised AGASACA using two manual counting methods.

To our knowledge, only one study had tried to determine a prognostic Ki67 cut off in dogs with anal sac tumors [17]. Only dogs with surgically excised small (≤3.2 cm) nonmetastatic AGASACA have been enrolled. Ki67 index has been determined in 20 samples and the mean value was evaluated in 3 non-consecutive HPF, in which 100 consecutive cells have been counted. There was no statistical difference in outcome for dogs with Ki67 <25% and ≥25%. In this study, the counting method described by Skorupski et al. (2018) was applied, but a different monoclonal mouse anti-human Ki67 antibody dilution (1:200) was used on a different AGASACA population. In our study the prognostic role of Ki67 was investigated in dogs with both anal sac tumor and lymph node metastasis, since dogs with regional metastasis at diagnosis showed a median DFI (283 days) significantly (*p* = 0.02) shorter than dogs without (940 days). As previously described, a correlation between the Ki67 index of the primary anal sac tumor and of the regional lymph node was found. Based on this finding, the authors decided to use a mean of the Ki67 values calculated for each dog for further determinations. Statistical analysis failed to prove the authors’ hypothesis that Ki67 index correlated with decreased DFI and ST in dogs with AGASACA with metastatic spread to regional lymph nodes.

Since of the aggressive behavior and the high metastatic rate of AGASACA are comparable to that of malignant oral melanoma, it was decided to count a greater number of histological fields to establish a diagnostic threshold for Ki67 index. To this purpose, the method described by Bergins et al. (2011) was used [29].The median Ki67 labeling index for dogs with lymph nodes metastasis was 32.45%, but no significant correlations were found with both ST and DFI.

Regional lymphadenopathy at diagnosis is reported in 26–89% of dogs with AGASACA [3,7,8,9,10,11,12]. In our study, 68.58% of dogs had lymphadenomegaly detected on preoperative CT scan. Regional lymph nodes metastatic spread was histologically confirmed after surgical removal in all cases. The presence of lymph nodes metastases at presentation reduced ST and DFI in several studies, thus being considered a negative prognostic factor [10,11,12,14]. In one study [11] ST in dogs with and without lymphadenopathy at presentation was 432 and 846 days, respectively. These data are similar to that reported by Barnes and Demetriou (2017), where dogs with and without lymph nodes involvement had a median ST of 846 and 432 days, respectively [25]. In our study, the median ST for 11 dogs without lymph node involvement was 1237 days, greater than that described in the previously reported study but equal to that reported by Skorupski et al. (2018) in 34 dogs without regional lymph nodes metastases [17]. Even if not statistically significant, a marked difference in survival time was found between these 11 dogs and 24 dogs with lymph nodes involvement. It is possible that a significance could be reached with a greater number of enrolled dogs. Anyway, twenty-four dogs with metastatic lymph nodes had a significantly (*p* = 0.02) shorter median DFI (283 days) than the 11 dogs without lymph nodes metastasis (940 days).

Based on these findings, the authors decided to split the population of the present study into two groups (with or without lymph nodes metastasis), and to investigate the prognostic role of different clinical (hypercalcemia and tumor size) and histologic factors besides Ki67 (MC, tumor pattern, presence of tumor necrosis, lymphatic and vascular tumor invasion, inflammatory infiltration, anisocytosis and anisokaryosis) (see Table 4) in the group with the worst prognosis (dogs with regional metastatic lymph-nodes).

Hypercalcemia was one of the investigated factors, which prognostic role is still controversial in the literature. According to William et al. (2003) and more recently to Heaton et al. (2020), hypercalcemic dogs at diagnosis had a significantly shorter survival time than normocalcemic dogs [9,13]. However, a similar evidence was not found in other studies [7,10,11,15]. The presence of hypercalcemia (20%, 7/35) in this study was similar to that reported previously [7,10,11,13,15]. The results of this study did not clarify the prognostic role of hypercalcemia in AGASACA. Hypercalcemia halved ST when investigated on both the entire population and in the subgroup of dogs with metastatic lymph nodes. Anyway, these results were not statistically significant, although very close to significance (*p* = 0.054) in both cases, maybe due to the small number of dogs enrolled in the study; a greater number would have yielded significant results.

Tumor size has been significantly associated with outcome in several studies [9,11,12,14,15,16,17,18]. Different cut offs due to different methods of tumor size measurement were used. These could have influenced the role of this parameter on the outcome. In this study, the median value of the maximum dimension of the excised mass was not significantly associated with outcome (ST and DFI) in dogs with metastatic lymph nodes. Therefore, the authors decided to select, as Pradel et al. (2018), the third quartile value [14]. Dogs with AGASACA <5.25 cm with regional metastasis had a median ST significantly longer (877 days) than dogs with AGASACA ≥5.25 (220.5 days), but a statistical difference in DFI was not reached.

Referring to the histologic characteristics of the tumor, the histologic classification proposed by Pradel et al. (2018) was used [14]. In accordance with previous studies [14,26], the most common tumor histologic pattern was the solid one and it was associated with a significantly (*p* = 0.03) shorter DFI in dogs with lymph nodes metastasis at diagnosis (median DFI: 155/523 days). There was also a difference in survival time, but it was not significant (*p* = 0.32; solid pattern MST = 352 days, other pattern MST = 877 days).

Median MC in this study was calculated both on the entire canine population and on the subpopulation of dogs with metastatic lymph nodes, and it was not significantly associated with ST and DFI in any case. This is in accordance with the results obtained from Ki67 evaluation; therefore, the authors concluded that the determination of indexes of cell proliferation may not be a useful tool to predict prognosis in dogs with AGASACA.

Among the other histologic characteristics evaluated, only the presence of lymphatic and vascular intra-tumor invasion was significantly associated with shorter ST in dogs with lymph nodes metastases at presentation (if present = 366 days; if absent = 1120 days). However, a statistical difference was not reached in DFI. This partially mirrors the findings of previous studies in which median ST and median DFI were statically shorter in tumors with presence of lymphatic and vascular intra-tumor invasion [14,18].

The role of adjuvant chemotherapy is unclear, in particular because of the lack of standardized protocols among patients [7,8,9,10,11,12,13,18,19]. In the present study, chemotherapy was proposed to all dogs, but only in 23 cases owners accepted to pursue it. Most of the treated dogs had regional lymph nodes metastasis. A statistical difference in ST and DFI between dogs that received adjuvant chemotherapy and dogs that did not, was not found, but chemotherapy administration seemed to increase ST in dogs with lymph nodes metastasis at diagnosis (median ST: 465 vs. 877 days). Toceranib phosphate (Palladia^®^) was the most commonly used drug, due to its potential antitumor activity against AGASACA [13,20,21,22]. In a recent study on 36 dogs with AGASACA treated with toceranib alone or in combination with surgery, progressive disease was observed only in 31% of treated dogs. Toceranib treatment-associated ST and DFI were 434 and 313 days, respectively [13]. In the present study, 57.14% of dogs treated with surgery and toceranib (as first or second chemotherapeutic agent) experienced progressive disease, median ST and DFI were 877 and 283 days, respectively.

Limitations of this study are primarily referable to its retrospective nature that affected the availability of some follow-up information and precluded the standardization of treatment. The small number of enrolled cases limited the statistical power and precluded a multivariable analysis, but at the same time reflects the nature of this neoplasia in dogs. One important factor that could have biased the determination of the Ki-67 index was the subjective determination of the Ki-67 immunostaining. Further investigations are necessary to determine a Ki67 index using a computer-assisted image software that is more accurate and standardized compared to the manual counting.

In conclusion, the results of this study showed that the presence of regional lymph nodes metastasis at diagnosis in dogs affected by AGASACA significantly reduced DFI. Moreover, tumor size greater than 5.25 cm, the presence of lymphatic and vascular invasion and a solid pattern were associated with a shorter ST in the same canine population. Ki67 expression was not significantly associated with prognosis, therefore it could not be considered as a prognostic factor in this tumor type, while the role of hypercalcemia remained unclear.

## Figures and Tables

**Table 1:** Adjuvant treatments after the first surgical removal of AGASACA or in case of tumor recurrence.

Drug	Dogs Treated	Treatment Discontinued Because of Adverse Effects	Progressive Disease	Therapy after Progressive Disease			
				Toceranib	Metronomic	Toceranib + Surgery	Euthanasia
Toceranib phosphate	21	4	12	4	2	1	5
Carboplatin	1	0	1	1	-	-	-
Melphalan	1	0	1	-	-	1	-

**Table 2:** Oncologic outcome of the 35 dogs included in the study. Two groups were considered: dogs with primary tumor only (*n* = 11) and dogs with both primary tumor and metastatic regional lymph nodes (*n* = 24).

Group	N° Dogs	Progressive Disease	Death for Tumor Related Causes	Death for Unrelated Tumor Causes	Median ST (Days)	Median DFI (Days)
Anal sac tumor only	11	5	4	5	1237	940
Anal sac tumor + lymph node metastasis	24	17	14	8	545	283

**Table 3:** Histological features of the AGASACA tumors.

Histological Features	Category	N° of Dogs
Pattern	Solid	21
	Rosettes/tubular	11
	Papillary	3
Necrosis	Present	8
	Absent	27
Inflammatory infiltration	Yes	23
	No	12
Lympho-vascular invasion	Yes	21
	No	14
Cellular pleomorphism	Minimal	27
	Moderate	7
	Marked	1
Mitotic count (median)	<17	16
	≥17	19
Lymph node predominant pattern	Solid	16
	Rosettes/tubular	8
	Papillary	0

**Table 4:** Prognostic factors evaluated in dogs with AGASACA and metastatic lymph nodes.

Variable	Groups	N° Dogs	Median ST (Days)	*p*-Value	Median DFI (Days)	*p*-Value
Hypercalcemia	Y N	10 14	343.5 1094	0.054	263 329	0.68
Tumor size (median value of the longest histologic dimension)	<3 cm ≥3 cm	8 16	877 545	0.52	283 273	0.59
3^rd^ Quartile tumor size (median value of the longest histologic dimension)	<5.25 cm ≥5.25 cm	18 6	877 220.5	0.005	385 145.5	0.11
Median Ki67 (300 cells)	<35.08 ≥35.08	12 12	559 352	0.52	385 273	0.71
Median Ki67 (500cells)	<32.45 ≥32.45	11 13	559 448.5	0.41	314 283	0.75
Median mitotic count	<16.5 ≥16.5	12 12	877 352	0.053	283 273	0.81
Predominant primary tumor pattern	Solid Others	14 10	352 877	0.32	155 523	0.03
Necrosis	Presence Absence	7 17	282.5 559	0.18	221.5 376	0.93
Lymphatic and vascular intra-tumor invasion	Presence Absence	18 6	366 1120	0.02	273 354	0.32
Inflammatory infiltration	Presence Absence	18 6	559 379	0.66	283 322	0.60
Anisocytosis and anisokaryosis	Mild Moderate Marked	18 5 1	559 354	0.32	376 137.5	0.12

## Data Availability

Data is contained within the article.

## Appendix A

**Table A1:** Supplementary individual information of each case.

Dog	Sex	Breed	Age (Years)	HyperCa^2^	Surgery	Enlarged Regional Lymph Nodes	Chemotherapy	ST (Days)	DFI (Days)	Alive	Median Ki67 300 Cells	Median Ki67 500 Cells
1	F	Boxer	9	N	L	Y	N	385	385	N^	30.15	27.60
2	F	German Shepherd	9	Y	L	Y	Y	598	598	LFU	30.82	27.00
3	FS	Mixed	12	N	L	Y	Y	352	273	N^	35.82	31.90
4	F	Mixed	15	Y	R	Y	Y	213	170	N^	34.67	32.10
5	MN	Labrador retriever	12	N	R	Y	Y	1394	1394	N	37.98	33.50
6	MN	Labrador retriever	12	Y	L	Y	N	128	121	N^	37.67	33.80
7	FS	Mixed	12	N	B; left mass	Y	Y	1145	119	N	31.33	28.70
8	FS	Mixed	10	N	B; left mass	Y	Y	979	979	N	39.50	37.40
9	FS	Mixed	8	N	R	N	Y	1434	1434	N	34.33	27.00
10	FS	Labrador retriever	11	Y	R	N	N	1237	1237	N^	19.60	16.20
11	FS	Border Collie	11	N	L	Y	Y	701	701	N	30.50	27.00
12	FS	Cavalier King Charles Spaniel	10	N	R	Y	Y	627	62	N	39.33	36.70
13	FS	Mixed	14	N	B	N	Y	415	378	N^	23.15	21.00
14	FS	German Shepherd	11	N	R	Y	N	545	474	N^	32.30	34.50
15	FS	Mixed	9	N	R	Y	Y	160	99	N^	40.47	36.30
16	MN	Mixed	13	N	L	N	N	529	529	N	30.00	27.20
17	FS	Mixed	7	N	R	Y	Y	1094	918	N^	55.98	51.20
18	FS	Siberian Husky	13	N	L	N	Y	332	332	N	51.00	46.80
19	FS	Beagle	10	Y	R	Y	N	559	523	N^	23.50	19.30
20	FS	Border Collie	9.5	Y	R	Y	Y	161	155	N^	32.67	30,20
21	FS	Mixed	13	N	R	Y	N	228	0	N^	44.73	42.30
22	M	Mixed	12	N	B; right mass	Y	Y	163	120	N^	39.80	35.40
23	MN	Mixed	12	Y	R	N	N	523	523	N	43.30	40.20
24	M	Labrador retriever	11	Y	L	Y	Y	877	376	N^	35.50	32.80
25	M	Border Collie	10	N	R	N	Y	946	940	N^	32.33	28.40
26	FS	Basset Hound	7	Y	R	Y	Y	321	283	N^	44.80	43.70
27	FS	Mixed	11	N	L	N	Y	1073	913	Y	34.33	32.00
28	FS	Mixed	14	N	R	N	N	235	235	N	43.00	39.60
29	MN	Labrador retriever	12.5	N	B; left mass	Y	N	793	62	N	24.00	22.60
30	FS	Mixed	9	Y	B	Y	Y	121	0	N	30.45	26.10
31	MN	Labrador retriever	11	N	R	N	N	576	576	N^	23.60	20.60
32	FS	Labrador retriever	10	N	L	N	N	819	819	Y	27.30	24.60
33	FS	Mixed	9.5	Y	R	Y	Y	366	243	N^	30.97	28.40
34	F	Labrador retriever	13	Y	L	Y	Y	108	108	N	42.30	37.90
35	FS	Mixed	10	N	L	Y	Y	385	385	Y	23.15	19.80

F, female intact; FS, female spayed; M, male intact; MN, male neutered; L, left sacculectomy; R, right sacculectomy; B, bilateral sacculectomy, Y, yes; N, no; LFU, lost to follow up; N^ disease related tumor.
